# Exploring the predictive “psycho-biomarkers” for checkpoint immunotherapy in cancer

**DOI:** 10.3389/fimmu.2025.1590670

**Published:** 2025-07-21

**Authors:** Qian Zuo, Jieting Chen, Xi Xiao, Yan Dai, Liushan Chen, Yuqi Liang, Yingchao Wu, Junfeng Huang, Rutao Cui, Rui Xu, Qianjun Chen

**Affiliations:** ^1^ Department of Breast, Guangdong Provincial Hospital of Chinese Medicine, Guangzhou, China; ^2^ State Key Laboratory of Traditional Chinese Medicine Syndrome, The Second Affiliated Hospital of Guangzhou University of Chinese Medicine, Guangzhou, China; ^3^ Guangdong Academy of Traditional Chinese Medicine, Guangzhou, China; ^4^ Department of Oncology, The First Affiliated Hospital of Guangzhou University of Traditional Chinese Medicine, Guangzhou, China; ^5^ The Second Clinical College of Guangzhou University of Chinese Medicine, Guangzhou, Guangdong, China; ^6^ School of Medicine, Zhejiang University School of Medicine, Hangzhou, China; ^7^ Chinese Medicine Guangdong Laboratory (Hengqin Laboratory), Zhuhai, China

**Keywords:** psychological stress, psycho-biomarkers, breast cancer, immunotherapy efficacy, predictive model

## Abstract

**Background:**

In recent decades, cancer immunotherapy has transformed the treatment landscape, offering significant advantages over traditional therapies by improving progression-free survival (PFS) and overall survival (OS). However, immune checkpoint inhibitors (ICIs) treatment has been associated with an increased risk of mortality in its early stages. Therefore, identifying reliable biomarkers to predict which patients will benefit clinically from ICIs therapy is critical. Depression, a common form of chronic psychological stress, has emerged as a regulator of tumor immunity and is gaining attention as a target for novel cancer treatments. To date, no studies have explored the potential of depression-related genes in predicting response to ICIs therapy.

**Methods:**

Public datasets of ICIs-treated patients were obtained from the TCGA and GEO databases, followed by comprehensive analyses, including bulk mRNA sequencing (mRNA-seq), co-expression network construction, and Gene Ontology enrichment. Regression analysis, using Cox proportional hazards and least absolute shrinkage and selection operator (Lasso), identified eight depression-related genes to build a predictive model for clinical outcomes in ICIs therapy. Additionally, correlations were explored between the depression-related predictive score and clinical parameters, including tumor mutational burden (TMB) and immune cell infiltration, establishing the score as a potential predictor of ICIs response.

**Results:**

The model categorized patients into high- and low-responsiveness groups, with significant differences in disease-free survival (DFS) between them. Validation using both internal and external datasets demonstrated the model’s strong predictive accuracy. Further analysis revealed that this response stratification correlates with immune cell abundance and TMB in cancer patients.

**Conclusion:**

This study suggests that depression-related genetic traits could serve as biomarkers for ICIs therapy response, tumor mutations, and immune system alterations. Our findings offer insights into personalized therapeutic strategies for early intervention and prognosis in specific cancer types.

## 1 Introduction

The global cancer burden is projected to reach 28.4 million cases by 2040, representing a 47% increase compared to 2020 (1). Cancer remains one of the most critical global health challenges. For decades, conventional treatments have relied on chemotherapy and ionizing radiation to target and reduce tumor mass. However, recent advancements in cancer immunotherapy have revolutionized the treatment of both solid and hematologic malignancies (2). The theoretical foundation for cancer immunotherapy emerged from the discovery of ‘immune checkpoints’—pathways that allow tumors to manipulate immune self-tolerance, evade immune detection, and escape immune surveillance (3). The development of immune checkpoint inhibitors (ICIs) through monoclonal antibodies, designed to prevent immune evasion, has spurred significant progress in immuno-oncology. In 2011, the US Food and Drug Administration (FDA) approved the first ICIs targeting cytotoxic T-lymphocyte-associated protein 4 (CTLA-4 or CD152) (4). Since then, inhibitors targeting programmed cell death protein 1 (PD-1 or CD279) and its ligand (PD-L1 or CD274) have gained approval for a broadening array of solid tumors, including melanoma, renal cell carcinoma (RCC), non-small cell lung carcinoma (NSCLC), and urothelial carcinoma, among others (5–8). Despite promising outcomes in responding patients, the early stages of ICIs therapy have been associated with an increased risk of mortality, as reflected in mortality curves (9). Potential explanations for this phenomenon include primary resistance, leading to accelerated tumor progression, and immune-related adverse events. Thus, determining whether ICIs therapy benefits individual patients is of paramount importance.

Extensive research has been conducted on pre-treatment biomarkers—biological indicators capable of reliably predicting clinical benefit in advance. Several factors have been identified as influencing the effectiveness of ICIs therapy, including age, viral status, tumor mutational burden (TMB), chemotherapy, antibiotic use, PD-L1 expression, epidermal growth factor receptor (EGFR) status, granulocyte-macrophage colony-stimulating factor (GM-CSF), and geographic heterogeneity (10, 11). Despite substantial efforts, only three biomarkers have received FDA approval for clinical use: tumor tissue PD-L1 protein expression, TMB, and mismatch repair (MMR) deficiency (12). These biomarkers are entirely dependent on access to tumor tissue for genomic testing and immunohistochemical staining. Moreover, the predictive value of these biomarkers can vary based on the therapeutic regimen. For instance, the CA209–538 clinical trial of combined anti-PD-1/CTLA-4 blockade therapies suggested that TMB’s predictive value in monotherapy might not be relevant in the context of combination therapies (13). Recent evidence also highlights host-related factors—such as smoking status (10), obesity (14), alcohol consumption (15), and psychological disorders (16)—as potential predictors of how a patient’s cancer responds to ICIs. Notably, modifying certain factors could offer opportunities for co-therapy strategies to extend the efficacy of ICIs treatment (17, 18).

Accumulating investigations have indicated that host-related characteristics such as emotional disorders are crucial players in cancer management (19). Psychological stress, a core component of emotional disorders, is a key feature of conditions like anxiety disorders, depression, and post-traumatic stress disorder. It can manifest depression, anxiety, sadness, and even physical symptoms (20). Serious health challenges, including a diagnosis of cancer, are recognized as precipitants of psychological stress, which is highly prevalent among patients with malignancies. For instance, among individuals with breast cancer, the reported prevalence of depressive and anxiety symptoms ranges from 32.2% to 41.9%, respectively (21, 22). Notably, the incidence of psychological stress in patients with cancer is estimated to be nearly fourfold higher than that in the general population (23, 24).

Psychological stress is also significantly associated with prognosis and survival in patients with cancer, as the presence of depressive or anxiety symptoms has been linked to an increased risk of disease recurrence and elevated mortality (25–27). Despite its clinical significance, the screening and monitoring of psychological stress are not routinely incorporated into contemporary medical practice (28). With the impressive anti-tumor activity and durable clinical benefits in diverse malignancies, elucidating the relationship between psychological stress and the ICIs efficacy has become increasingly important. To explore the potential role of psychological stress in ICIs outcomes, we introduced the TCGA and GEO databases to obtain intersected genes related to depression and ICIs efficacy. Through comprehensive bioinformatics analysis, a prognostic model for ICIs efficacy was developed based on depression-related genes. ICIs recipients were categorized into high-response and low-response subtypes according to their predicted therapeutic response. Further validation confirmed that this model serves as a reliable, independent indicator of immune response. Additionally, depression-related features were found to correlate with changes in clinicopathological factors and gene mutations. These findings may illuminate the potential relationship between depression-related genes and ICIs efficacy across various malignancies, offering novel insights into pre-treatment features for identifying ICIs responders.

## 2 Materials and methods

### 2.1 Sources of data, pre-processing, and training set profile

Clinical follow-up information and RNA sequencing data from patients with cancer were obtained from The Cancer Genome Atlas (TCGA, https://portal.gdc.cancer.gov/) and the Gene Expression Omnibus (GEO) database using the ‘TCGAbiolinks’ R package (29). Data preprocessing involved the following steps: (i) conversion of FPKM data to TPM format; (ii) removal of genes whose expression level was 0 in more than half of the samples; (iii) conversion of Ensembl IDs to gene symbols, where the median expression value was used for gene symbols with multiple corresponding Ensembl IDs; and (iv) log_2_ transformation of expression profile data.

ICBatlas compiled transcriptome and clinical data from ICIs-treated patient samples sourced from multiple databases, including the Gene Expression Omnibus (GEO), ArrayExpress, TCGA, and dbGaP. The dataset includes transcriptome features of ICIs therapy derived from 1,515 ICB-treated samples across 25 studies and 9 types of cancer. Samples were initially classified into response/nonresponse groups in ICBatlas. In this study, 4,782 differentially expressed genes (DEGs) were identified as transcriptome features of patients who received clinical benefits from ICIs therapy.

The 3539 Depression-related genes were retrieved from three databases: NCBI, GeneCards, and GSEA. From GeneCards, these genes with a relevance score greater than 1.0 were selected for further investigation. Additionally, the Molecular Signatures Database (MSigDB) was queried using the keyword “depression,” and seven depression-annotated gene sets were included for analysis. Furthermore, three depression-related datasets were retrieved from NCBI (using the keywords “depression” and “Homo sapiens”), and differential expression analysis between depression patients and control samples was conducted using the GEO2R tool to identify depression-related genes.

**Figure 1:**
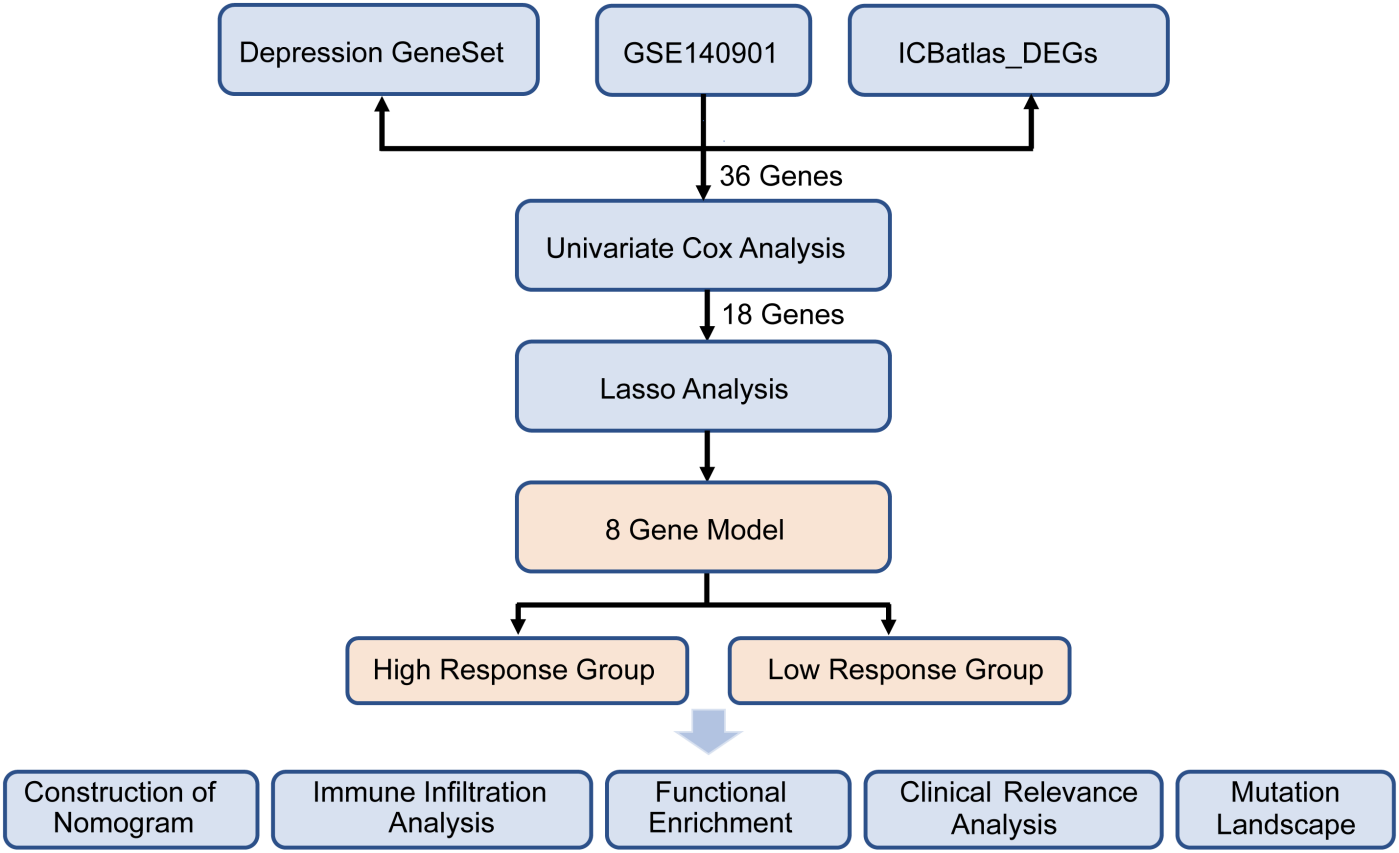
The flowchart of this study.

A comprehensive analysis was conducted on the 867 intersected genes in purple circle, and a volcano plot was used to display the number and distribution of DEGs (|log_2_FC| > 2, *p* < 0.05). Heatmaps of DEGs were generated using the heatmap (v1.0.12) package. DEGs were then subjected to gene function enrichment analysis using the Gene Ontology (GO) and Kyoto Encyclopedia of Genes and Genomes (KEGG) pathways databases, with statistical significance assigned to *p*-values < 0.05. The protein–protein interaction (PPI) network was constructed using Cytoscape software. Significant genes identified from univariate Cox regression analysis, based on response status, were visualized in a forest plot, and a gene correlation network was constructed using the survminer [v0.4.9] and igraph [v2.0.3] packages. The analytical flow of the study is presented in **Figure 1**.

### 2.2 The construction, evaluation, and validation of the predictive model

Patients in GSE140901 and GSE176307 were classified according to their clinical response. Responders were defined as those meeting the response evaluation criteria in solid tumors (RECIST) for either a full or partial response, or stable disease with progression-free survival (PFS) lasting more than six months. Non-responders were those with progressive disease or stable disease with PFS of fewer than six months, based on RECIST criteria.

For univariate Cox analysis, the coxph function from the R survival [v3.7-0] package was utilized, with a threshold of *P* < 0.05 applied for filtering. Subsequently, LASSO regression was performed using the R package ‘glmnet’ [v4.1-8] to refine the results of the univariate Cox regression. From the training datasets, LASSO regression identified eight depression-related genes associated with PFS in cancer patients. These eight genes, which were linked to both depression and ICIs therapy response, were further analyzed using multivariate Cox regression to establish a predictive model. The predictive model was constructed using the following formula:


$$RiskScore=\sum_{i=1}^{n}coef(i)\times gene(i)$$

where coef(i) represents the coefficient for gene i, and gene(i) denotes the expression level of gene i. The predictive formula derived from the coefficients of 8 genes is as follows: responsive score = (-2.13444) * (CD244 expression) + 1.26592 * (CMA1 expression) + (-0.78558) * (CSF1 expression) + 1.05026 * (FCGR2B expression) + 1.03112 * (IFNA1 expression) + (-3.27085) * (IL10 expression) + (-0.43313) * (SPP1 expression) + (-0.38439) * (TAP1 expression). To visualize the results, the ggrisk package [v1.3] was used to create scatter plots showing high- and low-response patients, along with their PFS times and survival statuses. A heatmap of the expression levels of the eight depression-related predictive genes was also generated. For external validation, patients from external datasets were categorized into high-response and low-response groups based on the median value of all samples. Kaplan-Meier survival curves were constructed to evaluate PFS, and log-rank tests were conducted to assess statistical significance across the training cohort (GSE140901), the external validation cohort (GSE176307), and the combined cohort (GSE140901 + GSE176307). Additionally, to assess the model’s performance, the area under the curve (AUC) of receiver operating characteristic (ROC) curves was calculated to determine the accuracy of the model in predicting PFS at 1, 3, and 5 years across these three datasets.

### 2.3 Analysis of the relationship between predictive models and other clinical features

To further validate the model’s prognostic value in relation to various clinical attributes and ICIs treatment response, univariate analysis was performed to explore the correlation between the predictive score and patients’ PFS. A nomogram was then developed, incorporating the responsive score along with clinical parameters such as age, gender, pathological stage, TMB, and PD-1 expression, using the rms package [v6.8-2] as independent prognostic factors. The precision of the nomogram was evaluated through decision curve analysis (DCA), which assessed the overall benefit of using the nomogram and clinical features separately. Additionally, ROC curves for PFS probabilities at 1 and 3 years were generated using the pROC package [v1.18.5] to further evaluate model accuracy.

### 2.4 Immune correlation analysis

Immune cell infiltration profiles were evaluated using the Xenophanean dataset, with immune cell infiltration assessed through EPIC, CIBERSORT, IPS, MCPCOUNTER, QUANTISEQ, TIMER, and XCELL algorithms within the IOBR [v0.99.0] package. Stromal, immune, and estimate scores, along with tumor purity, were calculated for high-response and low-response groups using the estimate [v1.0.13] package and the ssGSEA algorithm. The expression levels of genes associated with ICIs, extracted from published literature, were analyzed to compare variations between the high- and low-response groups. All statistical analyses and visualizations were performed using R software (v4.4.1), with statistical significance set at *P* < 0.05.

### 2.5 Analysis of differences between high and low responses of patients

To investigate potential causes of ICIs resistance in low-response patients, two groups were formed, and DEGs were identified using DESeq2 [v1.44.0]. GO, KEGG, and GSEA enrichment analyses were performed on the DEGs using the clusterProfile [v4.12.6] package. A heatmap of GSEA-enriched pathway scores was generated using pheatmap. Expression differences were analyzed with the GSEA algorithm to assess pathway enrichment disparities between groups, with pathways having *P* < 0.05 considered statistically significant.

### 2.6 Mutational landscape and the model of TMB predictive ability

To assess the predictive ability of TMB, data were gathered from a bladder cancer cohort (ICIs-treated BLCA, N = 455) undergoing immunotherapy, which included mutation, expression, and immunotherapy prognosis data from the IMvigor210CoreBiologies R package. Gene mutation patterns were extracted from the TCGA database and visualized using the maftools [v2.20.0] software. The depression-related predictive score was integrated with the gene mutation data, and variations in these genes within the cancer population were calculated. The association between the predictive model and TMB was assessed and visualized using ggpubr [v0.6.0].

### 2.7 Statistical methods

Group comparisons were conducted using Student’s t-tests (*P < 0.05, **P < 0.01, ***P < 0.001).

## 3 Results

### 3.1 Screening of predictive genes of immunotherapy efficacy related to depression

**Figure 2:**
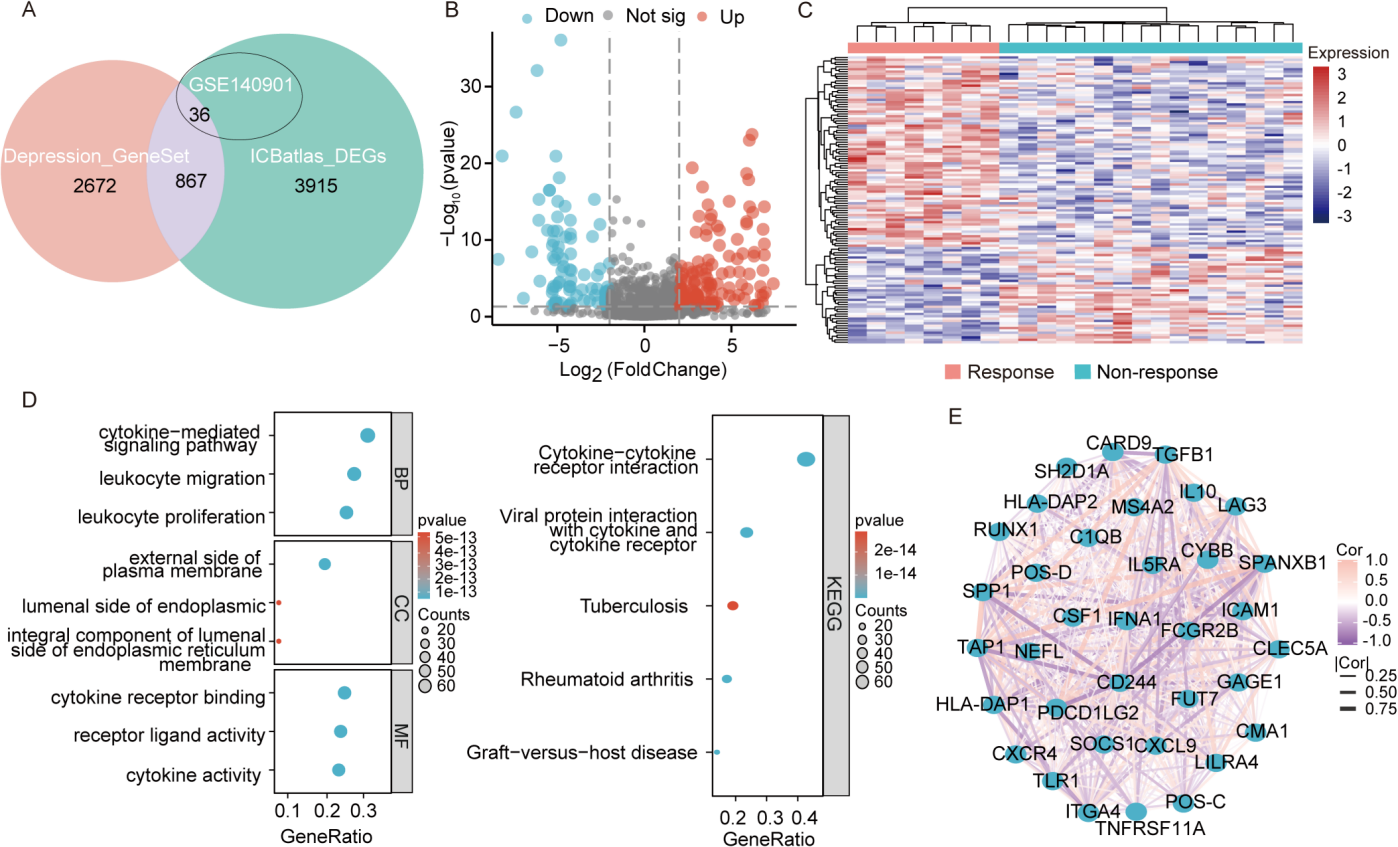
Transcriptomic analysis of DEGs in GSE140901. **(A)** The intersection of genes obtained in depression geneset, ICBatlas-DEGs, and GSE140901 database. **(B)** Differentially expressed genes (DEGs) volcano plot of the 122 DEGs in GSE140901, red represents significantly upregulated genes, blue represents significantly downregulated genes, grey represents genes with non-significant differences, the horizontal axis is log2Fold Change and the vertical axis is -log10q-value. **(C)** Heat map displaying the 122 DEGs created with the pheatmap R package (https://cran.r-project.org/bin/windows/base/old/4.1.3/). **(D)** GO and KEGG analysis of the 122 DEGs in GSE140901. **(E)** PPI network map of the 36 genes from the intersection of depression geneset, ICBatlas DEGs, and GSE140901 database.

**Figure 1** presents the flow diagram of the study. To obtain a comprehensive resource for characterizing ICIs therapy at the transcriptional level, ICBatlas was utilized to provide transcriptome features from the analysis of 1,515 ICB-treated samples across 25 studies spanning nine cancer types. As shown in **Figure 2A**, the green circle represents the 4,782 DEGs identified between response and non-response cohorts in ICBatlas, while the orange circle contains 3539 depression-related genes sourced from Genecards, NCBI, and GSEA databases (**Supplementary Table 1**). The transcriptional data from the hepatocellular carcinoma cohort treated with ICIs (GSE140901) was subsequently selected as the training set. Primarily, DEGs between different outcomes of ICIs therapy for HCC in GSE140901 were obtained. A total of 122 DEGs with statically significance were identified, with 81 genes upregulated and 41 downregulated (**Figures 2B, C**; **Supplementary Table 2**). To validate their potential roles in immunotherapy efficacy, GO enrichment and KEGG pathway analyses were performed on the 122 DEGs. The results revealed cytokine-cytokine receptor interaction were intensively enriched in KEGG analysis, and several GO terms related to the regulation of leukocytes and cytokines, which were closely involved in modulating the tumor microenvironment (TME) (**Figure 2D**). A protein–protein interaction (PPI) network was constructed to investigate the potential mechanism underlying depression and immunotherapy response. **Figure 2E** illustrated the protein–protein interaction (PPI) network consisting of 36 genes from GSE140901, which are also intersected with the 867 overlapping genes in **Figure 2A**. This analysis exhibited several cytokines and receptors, including IL10, CXCL9, and CXCR4, as key components in the relationship between depression and immunotherapy efficacy. Collectively, these results suggest that these cytokines may play a pivotal role in influencing the efficacy of immunotherapy.

### 3.2 Construction and validation of depression-related predictive model for immunotherapy efficacy

**Figure 3:**
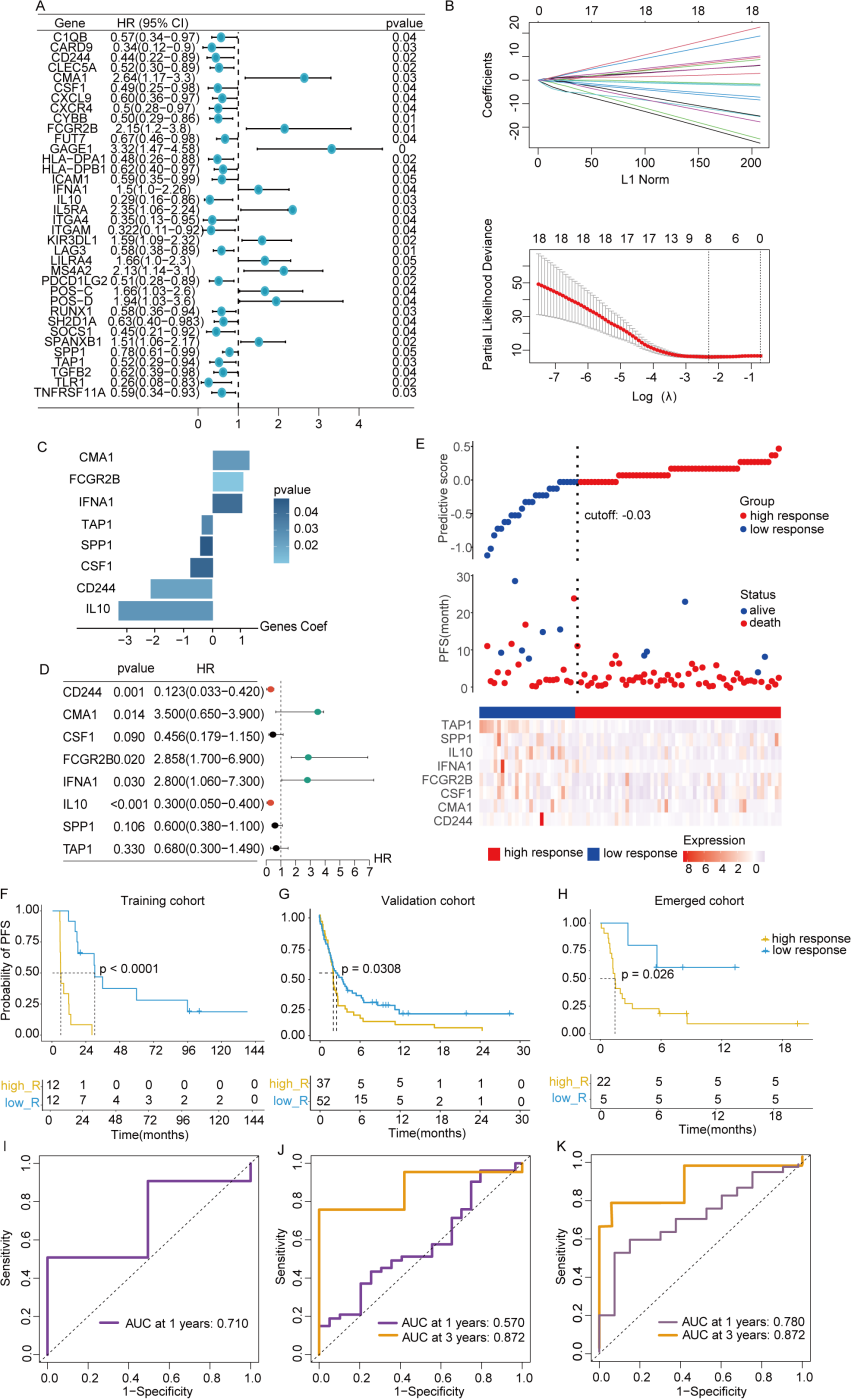
Construction of predictive signature. **(A)** Unitivariate Cox analysis was performed on the 36 intersected genes from the intersection of depression geneset, ICBatlas-DEGs, and GSE140901 database. **(B)** LASSO regression analysis based on the 36 intersected genes to develop the predictive model. **(C)** The 8 genes that ultimately built the signature. **(D)** Hazard ratios of the 8 model genes sourced from LASSO. **(E)** Variations in disease-free survival status and the expression levels of the 8 genes between groups with high and low responsive rates. **(F–H)** Values of AUC for the TCGA train, test, and full cohort. **(I–K)** Analysis of survival within the TCGA train, test, and full cohort. *P<0.05, **P< 0.01, ***P<0.001, ns indicates No significance.

To explore the relationship between depression and the clinical benefits of immunotherapy in patients, the training dataset from GSE140901 was utilized to construct the model. A total of 36 intersected genes from GSE140901, depression geneset and ICBatlas DEGs were collected to perform univariate Cox analysis, with identification of 18 predictive genes (**Supplementary Table 1**). A LASSO-Cox regression was conducted to address collinearity among these genes (**Figures 3A, B**). A multivariate stepwise regression analysis refined this list, ultimately selecting 8 genes for model development (**Figure 3C**). Corresponding predictive formula was derived from the 8 genes and applied to calculate the scores for each sample, with median values used to classify patients into high-response and low-response groups. Among the 8 genes, 3 were identified as risk factors, while the remaining 5 were protective factors (**Figure 3D**). Gene expression levels for both groups were visualized *via* a heatmap, which also revealed a shorter PFS period in the low-response group (**Figure 3E**). Validation was performed using data from a cohort of metastatic urothelial cancer patients treated with immune checkpoint blockade (ICB) between 2014 and 2021. Predictive analysis across the training cohort (GSE140901), external validation cohort (GSE176307), and combined cohort (GSE140901+GSE176307) confirmed that the low-response group exhibited poorer prognosis in all cohorts (**Figures 3F–H**). ROC curve analysis further assessed the accuracy of the depression-related gene signature in predicting immunotherapy efficacy, showing notable differences in AUC values at one year between high- and low-response groups across the training, test, and overall cohorts: 0.71, 0.57, and 0.78, respectively. At three years, the training cohort displayed an AUC of 0.872 (**Figures 3I–K**). These results support the robustness of the 8-gene signature in predicting survival benefits from immunotherapy.

### 3.3 A nomogram’s construction and validation

**Figure 4:**
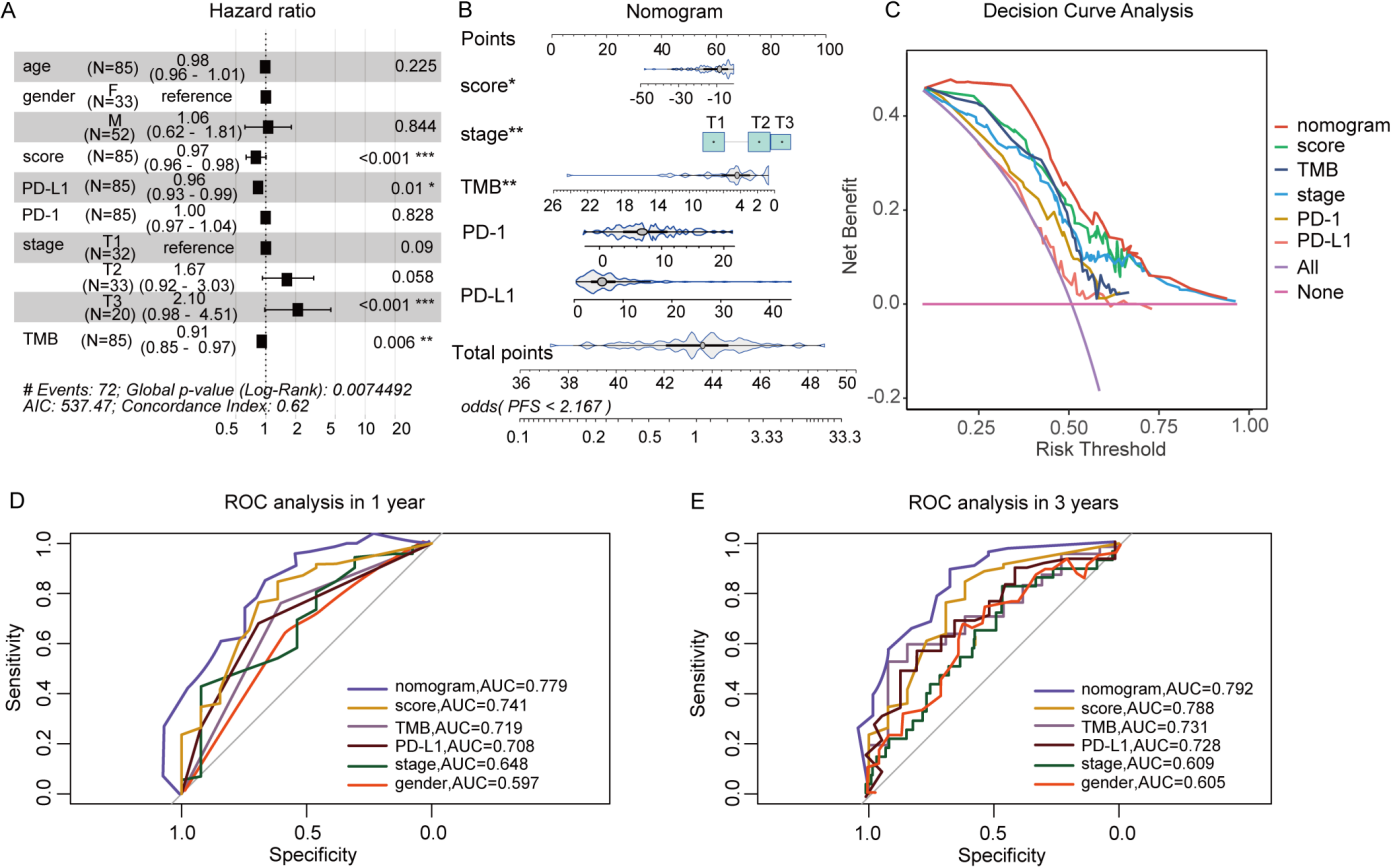
The Construction of nomogram based on predictive score and clinical factors. **(A)** Multivariate Cox analysis of responsive score and clinical factors. **(B)** Nomogram to predict the ICIs outcomes of patients with cancer. **(C)** Decision curve for the nomogram. **(D, E)** Nomogram’s 1- and 3-year disease-free survival time ROC curve, respectively. *P<0.05, **P<0.01, ***P<0.001.

To further evaluate the predictive model’s contribution relative to other biomarkers in assessing immunotherapy response, a nomogram was constructed. This nomogram serves as a clinical decision-support tool, assisting in identifying high-response patients for targeted therapies. Additionally, DCA was conducted to assess the clinical impact of various contributors by analyzing their AUC and the horizontal axis for no intervention. As shown in **Figure 4A**, predictive scores, PD-L1 expressions, and TMB were identified as protective factors, while advanced T stage emerged as a risk factor for poor ICIs efficacy. Nomogram analysis further demonstrated that the nomogram outperformed other clinical indicators in predicting patient response to immunotherapy, confirming its potential as an effective clinical decision-making tool (**Figures 4B, C**). ROC curve analysis revealed that the nomogram exhibited an AUC of 0.779 at 1 year and 0.792 at 3 years, indicating robust predictive accuracy (**Figures 4D, E**).

### 3.4 Distinction of immune landscape based on prediction score

**Figure 5:**
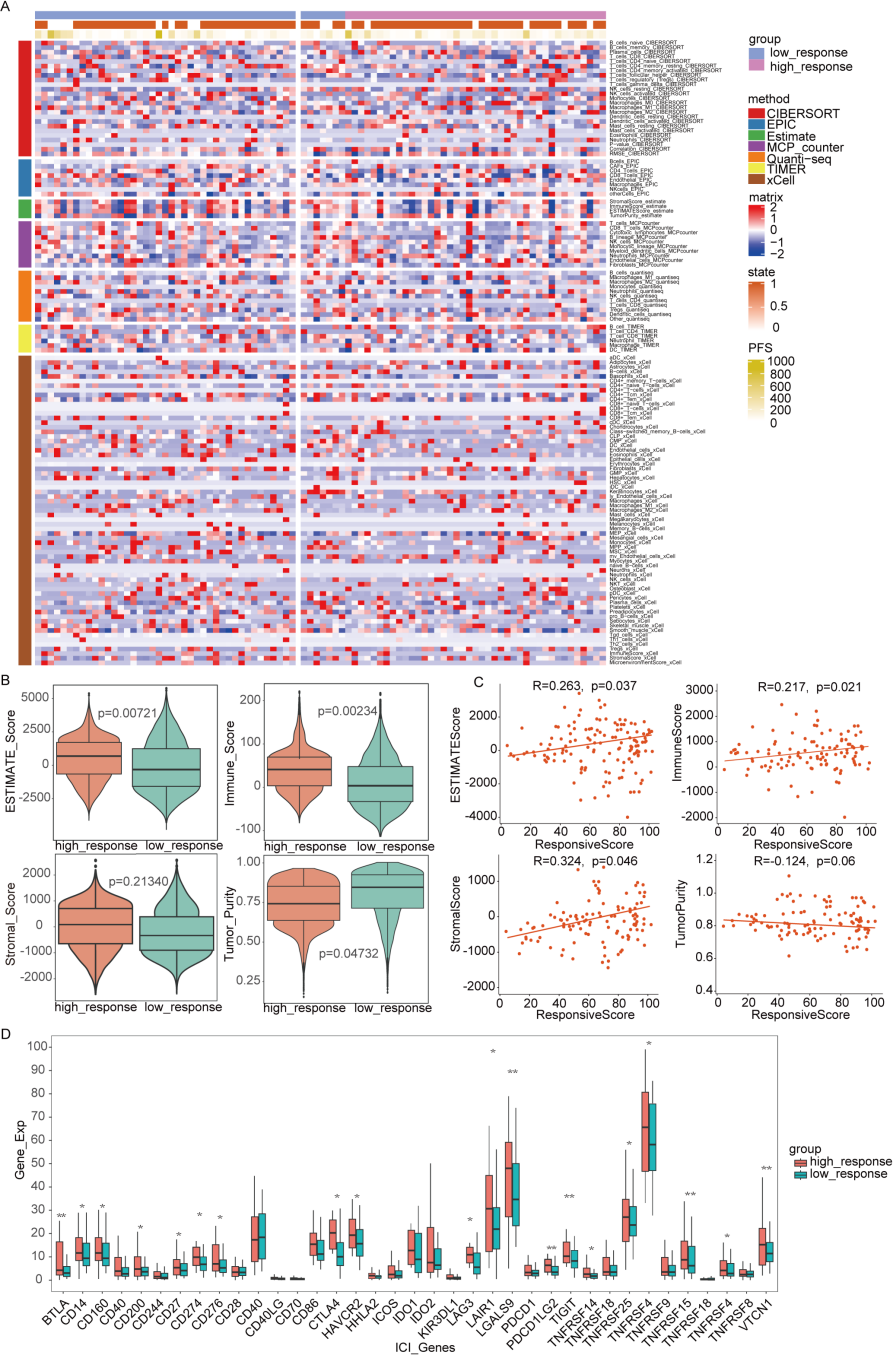
Immune infiltration analysis between two subtypes. **(A)** The full cohort’s distribution and correlation of the 22 tumor-infiltrating immune cells (TICs). **(B, C)** Analysis of the correlation between immune score and responsive score, ESTIMATE score and responsive score, stromal score and responsive score, tumor purity and responsive score. **(D)** Variations in the abundance levels related to immune-checkpoint-related genes between groups with high and low responses. *P<0.05, **P< 0.01, ***P<0.001.

To deepen our understanding of the tumor immune microenvironment in both low- and high-response groups, several methods were employed to quantify immune cell infiltration levels. As illustrated in **Figure 5A**, the ssGSEA algorithm was used to compute enrichment scores of 24 immune cell types, revealing that the low-response group exhibited a reduced presence of key immune cells, including B cells, T cells, CD8+ T cells, cytotoxic cells, dendritic cells, and mast cells. Using the TIMER and EPIC algorithms, which calculated immune cell enrichment scores for six and seven immune cell types, respectively, both analyses confirmed a lower abundance of CD4+ T cells and B cells in the low-response group. The low-response group was characterized by markedly reduced immune infiltration, whereas the high-response group showed decreased tumor purity but higher ESTIMATE and stromal scores (**Figures 5B, C**). These results underscore the relationship between immune infiltration and varying responses to immunotherapy. Additionally, with increasing focus on immunotherapy targeting immune co-suppressor molecules, the expression levels of several immune checkpoints were examined across both response groups. As depicted in **Figure 5D**, the high-response group exhibited elevated expressions of BTLA, LGALS9, PDCD1LG2, TIGIT, TNFSF15, and VTCN1 compared to the low-response group, suggesting that patients with this tumor profile may benefit from ICIs therapy.

### 3.5 Identification of differentiated expressed genes and analysis of functional enrichment across two subtypes

**Figure 6:**
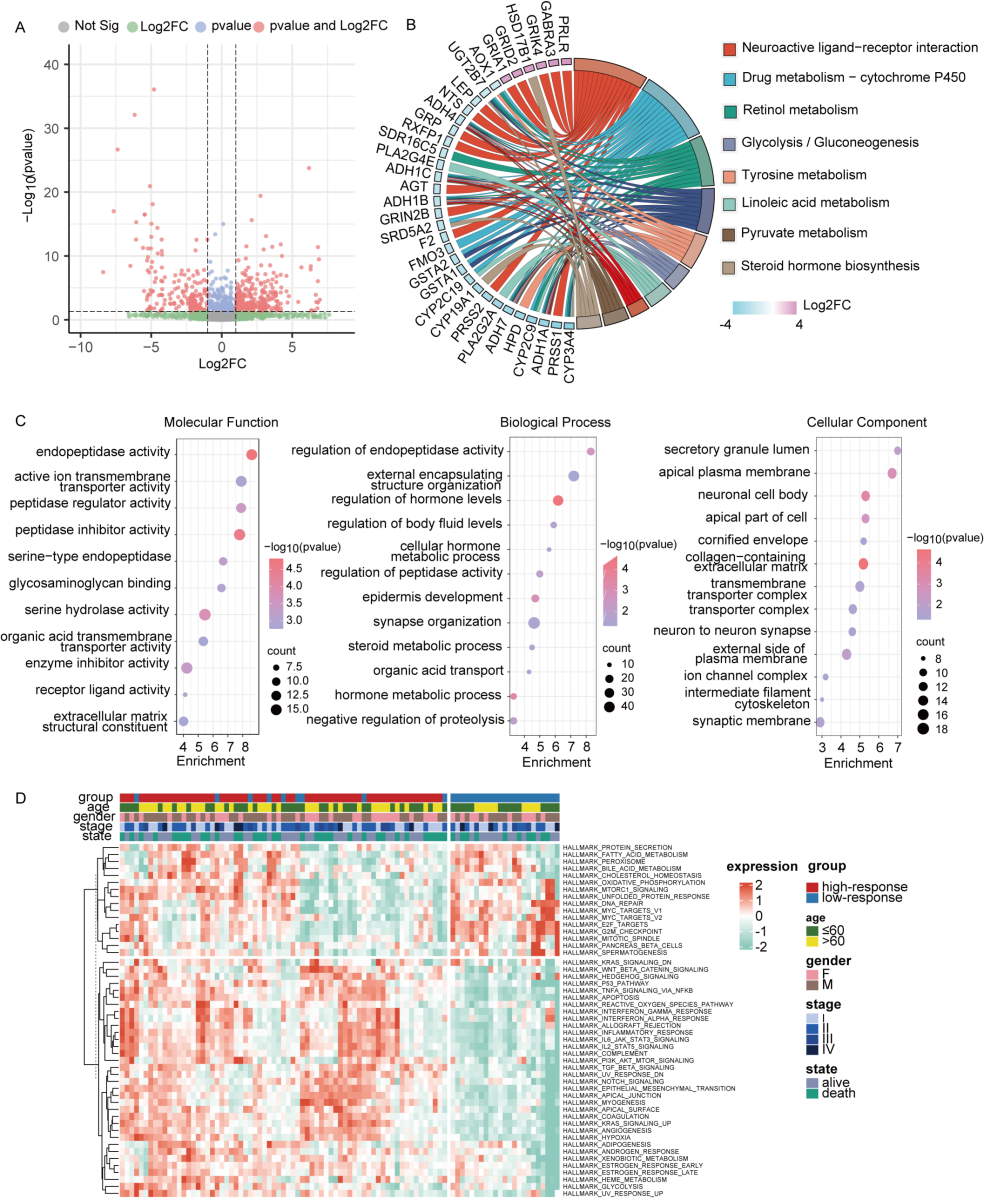
Analysis of functional enrichment across two subtypes. **(A)** Genes that were expressed differently between two subtypes in the entire cohort were displayed on the volcano map. **(B)** Differentially expressed genes were selected for KEGG analysis (|FC|>2, p < 0.05). **(C)** GO analysis of differentially expressed genes (|FC|>2, p < 0.05). **(D)** Differences in GSVA scores between two subtypes were displayed by heat map. *P<0.05, **P< 0.01, ***P<0.001.

A total of 310 up-regulated and 275 down-regulated genes were observed among the two response groups in **Figure 6A** (Fold change>2, pvalue<0.05). KEGG and GO analyses were then conducted based on these 585 genes. KEGG analysis highlighted the enrichment of DEGs in neurobiological and metabolic pathways (**Figure 6B**), including neuroactive ligand-receptor interactions, tyrosine metabolism, glycolysis, and linoleic acid metabolism. GO enrichment analysis, as depicted in **Figure 6C**, identified relevant biological processes, cellular components, and molecular functions. Additionally, the GSVA algorithm was utilized to assess variations in biological pathways between the two groups with differing immunotherapy responses. A total of 50 statistically significant biological pathways were identified based on the contrasts in GSVA scores between the groups (**Figure 6D**). These results suggest that the low-response group is significantly linked to neurobiological and metabolic functions, which may contribute to poor immunotherapy efficacy.

### 3.6 Clinicopathological and mutation landscape analysis of the predictive signature

**Figure 7:**
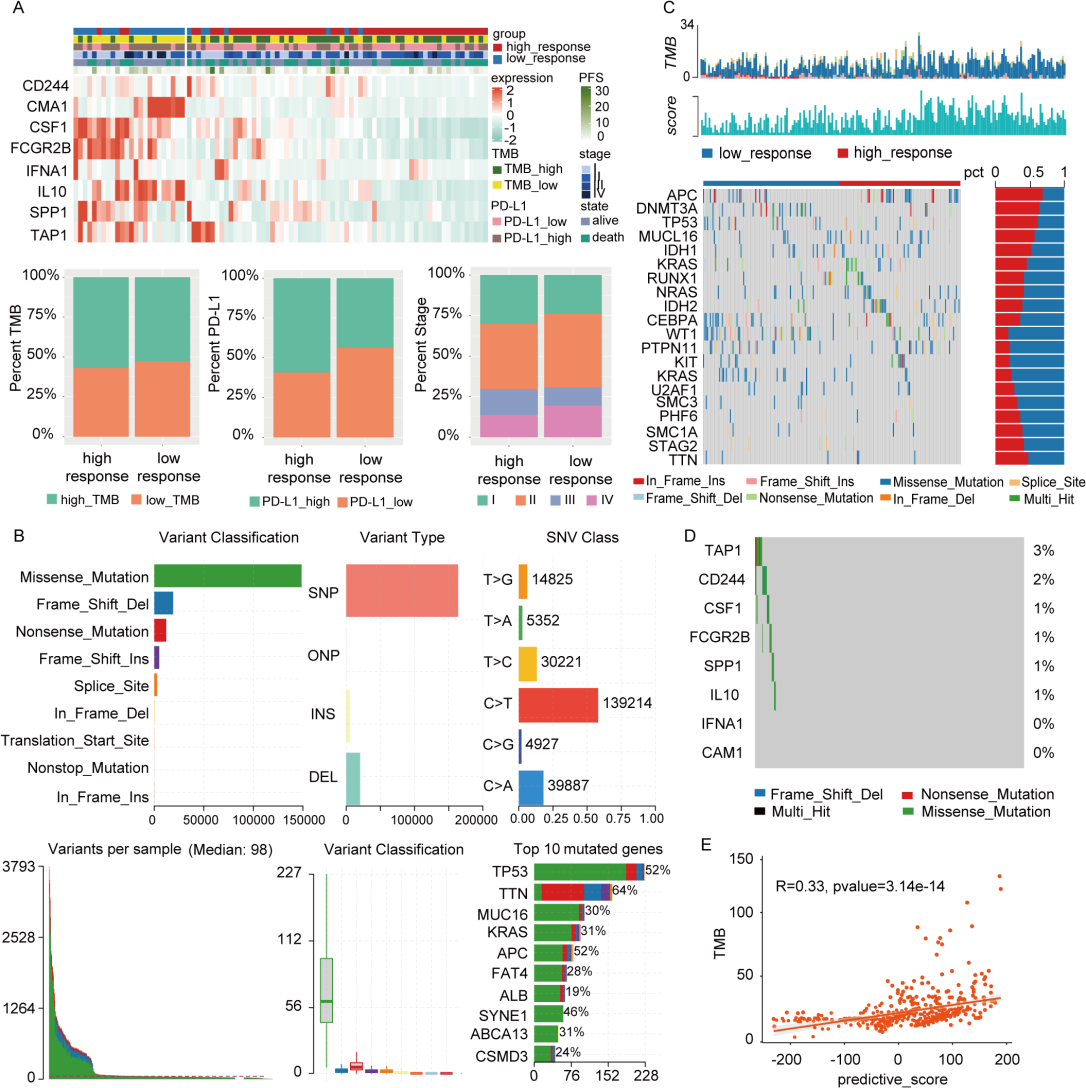
Clinical relevance and gene mutation analysis between two subtypes. **(A)** There were significant differences in PD-L1 expression, TMB status, N stage, and progression-free survival between groups with high and low responses. **(B)** Mutation landscape in full cohort, including variant classification, variant type, SNV class, variants per sample, variant classification summary, and top 10 mutated genes. **(C)** The representative gene mutations of the two subtypes. **(D)** The mutations of 8 model genes. **(E)** The correlation between TMB and responsive score.

To explore clinical attributes differentiating the two response groups, a clinical heatmap was generated. As shown in **Figure 7A**, the high-response group had a higher proportion of patients with elevated TMB and PD-L1 expression compared to the low-response group. This observation aligns with the predictive value of established biomarkers and reinforces their utility in forecasting the clinical benefits of immunotherapy in depressed patients.

Mutation analysis is summarized in **Figure 7B**, with missense mutations being the predominant mutation type. The top three genes with the highest mutation frequencies were TP53, TTN, and MUC16. Representative gene variants for each group were also assessed. In the low-response group, the most frequent mutations occurred in WT1, PTPN11, KIT, KRAS, and U2AF1, whereas in the high-response group, the highest mutation frequencies were observed in APC, DNMT3A, TP53, MUC16, and IDH1 (**Figure 7C**). **Figure 7D** details the mutations in 8 depression-related genes incorporated into the predictive model, including TAP1, CD244, CSF1, FCGR2B, SPP1, and IL-10. A significant difference in TMB levels between the two groups was observed, with risk scores exhibiting a positive correlation with TMB values (**Figure 7E**).

## 4 Discussion

Chronic psychological stress, particularly depression, is a major contributor to global health disorders and a common comorbidity in cancer, affecting more than 10% of patients (30). Accumulating evidence from animal models and human studies suggests that depression can activate either the hypothalamus-pituitary-adrenal (HPA) axis or the sympathetic nervous system (SNS), thereby influencing the initiation and progression of specific cancer types (31). Stress hormones and/or neurotransmitters, secreted systemically or locally released in the TME from activated sympathetic nerve endings, can enhance the malignant properties of cancer cells (32–34). Infiltrating nerves activated by depression can further influence various tumor biological processes through exosomes and neurotransmitters (35), thus reinforcing the clinical relevance of depression in cancer progression.

This study identified eight depression-related genes through Cox and Lasso regression analysis and developed a predictive model for immunotherapy response. Based on median risk values, patients were categorized into high and low-response groups. ROC curve analysis across the training, test, and full cohorts confirmed the model’s accuracy, with a maximum AUC value of 0.872 at 3 years. To compare the decision-making value of this model with other clinical characteristics in predicting ICIs outcomes, a nomogram was constructed by integrating responsive scores, TMB status, TNM stages, and tumor PD-L1 expression (36). The results demonstrated that responsive scores were more efficient than other clinical features in predicting immunotherapy effectiveness, offering a broader framework for identifying potential predictors of immunotherapy outcomes and highlighting the significance of “psycho-biomarkers” in cancer treatment (37).

The distinct clinical features and TME landscapes between the two response groups were further examined. A higher proportion of patients with high TMB and PD-L1 positivity was observed in the high-response group compared to the low-response group, suggesting the potential utility of the model in predicting patient responses to ICIs. Given the established link between depression and tumor immunity, ESTIMATE, TIMER, EPIC, and ssGSEA algorithms were employed to investigate the relationship between responsive scores and the TME. Our analysis revealed that higher responsive scores were significantly associated with an increased abundance of immune cells such as B cells, T cells, CD8+ T cells, cytotoxic cells, and dendritic cells. Additionally, the high-response group exhibited elevated expressions of immune co-inhibitory genes, including LAG3, PDCD1, CD274, and PDCD1LG2, indicating a low-affinity immune state in these patients. Previous studies have shown that T cells, when functionally exhausted over extended periods, co-express multiple co-inhibitory proteins, suggesting that targeting several checkpoints simultaneously or sequentially in high-response groups could elicit a stronger anti-tumor response (38). Notably, a positive correlation was also identified between TMB levels and responsive scores. Previous research has established TMB status as a predictive biomarker for identifying patients likely to benefit from ICIs treatments (39). Our findings support this, with responsive scores showing a positive correlation with TMB, consistent with prior results.

To further investigate depression-related genes central to modulate immunotherapy response, functional enrichment analysis was performed using the ssGSEA algorithm. The results indicated that the high-response group favored neurobiological and metabolic pathways, as evidenced by elevated enrichment scores for gene clusters involved in neuroactive ligand-receptor interactions, tyrosine metabolism, glycolysis, and linoleic acid metabolism. Abnormal metabolic activity is crucial in tumor progression, as supported by numerous studies (40, 41).

In contrast, the low-response group displayed activity in various pathways, including high enrichment scores for E2F targets related to G2M checkpoints, unfolded protein responses, MYC targets, oxidative phosphorylation, DNA repair, and other functions associated with tumor progression. Importantly, the high-response group showed significant enrichment in signaling pathways such as Wnt-β-catenin, p53, TNF-α, IFN-γ/α, IL6-JAK-STAT3, PI3K-AKT-mTOR, TGF-β, and Notch. Abnormal activation of these pathways plays a pivotal role in tumor cell growth, migration, and invasion, thus remodeling the TME and correlating with poorer prognosis (42–44).

The mechanisms of immune modulation associated with the eight predictive depression-related genes involve several key immune regulators. IL-10, CSF-1, and IFNα1 are active cytokines involved in immune responses (45–48). CD244, an inhibitory receptor primarily found on NK cells, T cells, and other immune cells, modulates their activation and function (49). FCGR2B, another inhibitory receptor, regulates the interaction between antibodies and immune cells, particularly B cells and CD8+ T cells (50). TAP1 is a crucial component of the TAP complex, responsible for transporting peptides to major histocompatibility complex (MHC) class I molecules for presentation to cytotoxic T cells (51). SPP1, secreted by tumor-associated macrophages (TAMs), enhances cancer cell migration and invasion, as demonstrated in A549 lung cancer cells (52). CMA1, a glycosyltransferase expressed in mast cells, may influence immune responses (53). Additionally, emerging studies suggest that psychological stress plays a significant role in tumor immunity (54). *In vivo* studies have demonstrated that depressed mice with cancer exhibit lower activation levels of T helper cells, leading to immune escape through immunosuppression (55, 56). Furthermore, depression-related nervous signaling has been shown to promote TAM growth in xenograft breast cancer models (57). As experimental research advances, clinical studies have increasingly explored the impact of depression on cancer development and treatment. Most clinical investigations focus on the epidemiology and risks associated with depression in cancer patients, particularly the strong correlation between severe depression and poorer clinical outcomes (24, 58, 59). A recent prospective observational study demonstrated that emotional distress (ED) significantly reduced the effectiveness of initial ICIs therapy in advanced non-small-cell lung cancer patients. The ED group had a 2-year overall survival rate of 46.5%, compared to 64.9% in the non-ED group, along with a decrease in quality of life. This study provides direct evidence of the negative impact of depression on immunotherapy efficacy, highlighting the potential role of psychological factors in cancer immunotherapy. Consequently, systematically exploring how depression-related genes predict immunotherapy effectiveness is of practical significance. Furthermore, this study offers a reference for future clinical trials, suggesting that ED could be considered a baseline characteristic in study designs.

### 4.1 Study limitations

Despite the promising results, several limitations must be acknowledged. First, our analysis was based on TCGA and GEO databases, and this study only examines the association between depression and ICIs outcomes in specific cancer types. Additional validation with larger clinical study cohorts is necessary to confirm the accuracy of our model. Second, the outcomes of immunotherapy vary depending on the regimen and drugs used, highlighting the need for future studies to assess the model’s efficacy across different treatment protocols. Lastly, depression in this study was inferred solely from molecular profiles rather than clinical psychometric data. The absence of clinical depression phenotyping, such as assessment with PHQ-9 scores, may introduce bias, particularly given the retrospective nature of data mining.

### 4.2 Clinical implications and conclusions

This study contributes to a deeper understanding of the role of “psycho-biomarkers” in cancer treatment, emphasizing the potential significance of the psychological dimension in cancer immunotherapy. The findings provide valuable insights into future clinical trials, suggesting that ED should be considered a baseline characteristic in study designs. Additionally, a detailed study design is included in the supplementary files (**Supplementary Figure 1**). Our results indicate that the model based on 8 depression-related genes is strongly correlated with ICIs therapy outcomes, underscoring the relevance of psychological factors in cancer immunotherapy. Further analysis revealed that this response grouping could offer a potential approach for evaluating immune cell abundance and tumor mutation burden in cancer patients.

## Data Availability

Publicly available datasets were analyzed in this study. This data can be found here: TCGA: https://portal.gdc.cancer.gov/; ICBatlas: https://guolab.wchscu.cn/ICBatlas/; GEO: https://www.ncbi.nlm.nih.gov/gds/.
