# Supplementary figures and images for: Exploring the predictive “psycho-biomarkers” for checkpoint immunotherapy in cancer
Source: Front Immunol. 2025 Jul 21;16:1590670. doi: 10.3389/fimmu.2025.1590670 (PMC12318748; doi:10.3389/fimmu.2025.1590670)

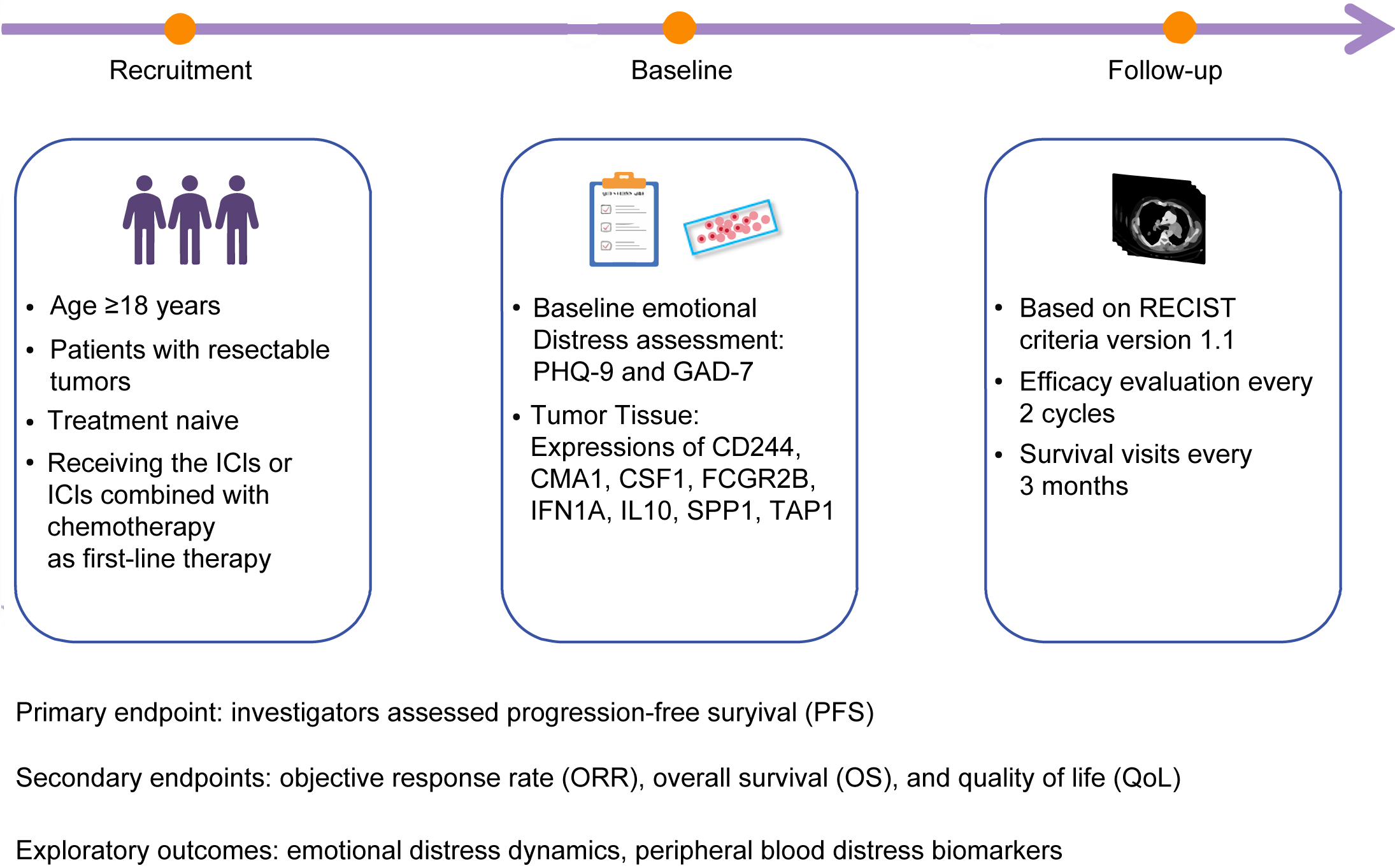

Supplement: Supplementary file 1 [file DataSheet1.zip › Supplementary file 1.TIF]
